# Transcriptomic, Protein-DNA Interaction, and Metabolomic Studies of VosA, VelB, and WetA in Aspergillus nidulans Asexual Spores

**DOI:** 10.1128/mBio.03128-20

**Published:** 2021-02-09

**Authors:** Ming-Yueh Wu, Matthew E. Mead, Mi-Kyung Lee, George F. Neuhaus, Donovon A. Adpressa, Julia I. Martien, Ye-Eun Son, Heungyun Moon, Daniel Amador-Noguez, Kap-Hoon Han, Antonis Rokas, Sandra Loesgen, Jae-Hyuk Yu, Hee-Soo Park

**Affiliations:** a Department of Bacteriology, University of Wisconsin—Madison, Madison, Wisconsin, USA; b Department of Biological Sciences, Vanderbilt University, Nashville, Tennessee, USA; c Biological Resource Center (BRC), Korea Research Institute of Bioscience and Biotechnology (KRIBB), Jeongeup-si, Republic of Korea; d Department of Chemistry, Oregon State University, Corvallis, Oregon, USA; e The Whitney Laboratory for Marine Bioscience, University of Florida, Gainesville, Florida, USA; f School of Food Science and Biotechnology, Kyungpook National University, Daegu, Republic of Korea; g DOE Great Lakes Bioenergy Research Center, University of Wisconsin—Madison, Madison, Wisconsin, USA; h Department of Pharmaceutical Engineering, Woosuk University, Wanju, Republic of Korea; i Department of Systems Biotechnology, Konkuk University, Seoul, Republic of Korea; University of Georgia

**Keywords:** sporulation, asexual development, velvet, WetA, secondary metabolites, *Aspergillus*, transcription factor, genetic regulatory network

## Abstract

In filamentous fungi, asexual development involves cellular differentiation and metabolic remodeling leading to the formation of intact asexual spores. The development of asexual spores (conidia) in *Aspergillus* is precisely coordinated by multiple transcription factors (TFs), including VosA, VelB, and WetA. Notably, these three TFs are essential for the structural and metabolic integrity, i.e., proper maturation, of conidia in the model fungus Aspergillus nidulans. To gain mechanistic insight into the complex regulatory and interdependent roles of these TFs in asexual sporogenesis, we carried out multi-omics studies on the transcriptome, protein-DNA interactions, and primary and secondary metabolism employing A. nidulans conidia. RNA sequencing and chromatin immunoprecipitation sequencing analyses have revealed that the three TFs directly or indirectly regulate the expression of genes associated with heterotrimeric G-protein signal transduction, mitogen-activated protein (MAP) kinases, spore wall formation and structural integrity, asexual development, and primary/secondary metabolism. In addition, metabolomics analyses of wild-type and individual mutant conidia indicate that these three TFs regulate a diverse array of primary metabolites, including those in the tricarboxylic acid (TCA) cycle, certain amino acids, and trehalose, and secondary metabolites such as sterigmatocystin, emericellamide, austinol, and dehydroaustinol. In summary, WetA, VosA, and VelB play interdependent, overlapping, and distinct roles in governing morphological development and primary/secondary metabolic remodeling in *Aspergillus* conidia, leading to the production of vital conidia suitable for fungal proliferation and dissemination.

## Abstract (precis)

Filamentous fungi produce a vast number of asexual spores that act as efficient propagules. Due to their infectious and/or allergenic nature, fungal spores affect our daily life. *Aspergillus* species produce asexual spores called conidia; their formation involves morphological development and metabolic changes, and the associated regulatory systems are coordinated by multiple transcription factors (TFs).

## INTRODUCTION

Fungal asexual spores are key reproductive cells that are essential for the long-term survival of filamentous fungi under a variety of environmental conditions (1). These spores can easily disperse into various environmental niches and act as infectious units for some pathogenic fungi (2–4). Asexual development in *Aspergillus* involves the formation of multicellular structures called conidiophores, each bearing hundreds of asexual spores called conidia. The production of intact conidia (conidiation) requires highly specialized cellular and structural differentiation and metabolic remodeling, which is governed by the coordinated activities of multiple positive and negative regulators (5, 6). Current knowledge about conidiogenesis is derived from numerous studies in model filamentous fungi such as Aspergillus nidulans (7–10).

The entire process of conidiogenesis is regulated by distinct gene sets, including central, upstream, and feedback regulators (6, 11). These components are highly conserved in *Aspergillus* species (12). In order to initiate conidiation, upstream developmental activators (FluG and FlbA, FlbB, FlbC, FlbD, and FlbE) induce the activation of *brlA*, an essential initiator of conidiation (13). This occurs when the fungal cells have acquired developmental competence that involves the removal of repressive effects imposed by the key negative regulators SfgA, NsdD, and VosA (14–16). Upon the activation of BrlA, it turns on AbaA and WetA, and together they sequentially control the conidiation-specific genetic regulatory networks, thereby governing the formation of conidiophores consisting of aerial stalks, vesicles, metulae, phialides, and conidia (9, 17). These three regulators are considered to form the central regulatory pathway (BrlA→AbaA→WetA) in *Aspergillus* species (18). BrlA is a key transcription factor (TF) that activates the expression of *abaA* and other genes in the early stage of conidiation (19, 20). AbaA is a TEF1 (transcriptional enhancer factor 1) family TF governing the expression of certain genes such as *wetA*, *vosA*, *velB*, and *rodA* in the metulae and phialides (21–23). WetA plays an important role in conidial wall integrity and conidial maturation during the late phase of conidiogenesis (24, 25). Our recent studies have shown that WetA functions as a DNA-binding protein that regulates spore-specific gene expression (25, 26). Along with WetA, two velvet regulators, VosA and VelB, which are fungus-specific TFs, coordinate morphological, structural, and chemical developments and exert feedback control of BrlA in conidia (27–30).

Previous studies have found that single-knockout mutants of *vosA*, *velB*, and *wetA* share multiple conidial phenotypes, including reduced spore viability, impaired trehalose biosynthesis, defective cell wall integrity, and reduced stress tolerance (25, 31, 32). The mRNA levels of these three regulators are high in wild-type (WT) conidia (25, 27, 28, 33). Results of chromatin immunoprecipitation (ChIP) analyses have demonstrated that VosA and WetA recognize certain DNA sequences in the promoter regions of target genes and regulate the mRNA expression of spore-specific genes in asexual spores (25, 29). In addition, the deletion of *vosA* or *wetA* affects the mRNA levels of multiple secondary metabolite cluster genes (25, 30, 34). Biochemical studies have determined that VosA interacts with VelB in conidia, and this complex controls trehalose and β-glucan biosynthesis (30, 35). Importantly, the roles of these three TFs are conserved in *Aspergillus* species (36–39). Considered jointly, these results suggest that VosA, VelB, and WetA are key TFs that orchestrate spore-specific gene expression in A. nidulans. Although the role of each regulator has been studied, the regulatory networks between these proteins have not, to date, been investigated in detail. In addition, the effects of these three proteins on primary and secondary metabolism are yet to be elucidated.

In this study, we aimed to determine the cross-regulatory mechanisms of VosA/VelB/WetA in fungal conidiation using comparative transcriptomic and metabolomic analyses of WT and null mutants of *wetA*, *velB*, and *vosA* in A. nidulans conidia. In addition, the direct targets of these regulators were identified by combining the results from the VosA- and VelB-chromatin interactions using ChIP sequencing (ChIP-seq) analysis with WetA direct targets identified in a previous study (25). The results clarify the detailed molecular mechanisms by which VosA/VelB and WetA control defined common and distinct regulons and increase the overall understanding of the regulatory networks that govern fungal cell differentiation and metabolism.

## RESULTS

### VosA-, VelB-, and WetA-mediated gene regulation in A. nidulans conidia.

**FIG 1:**
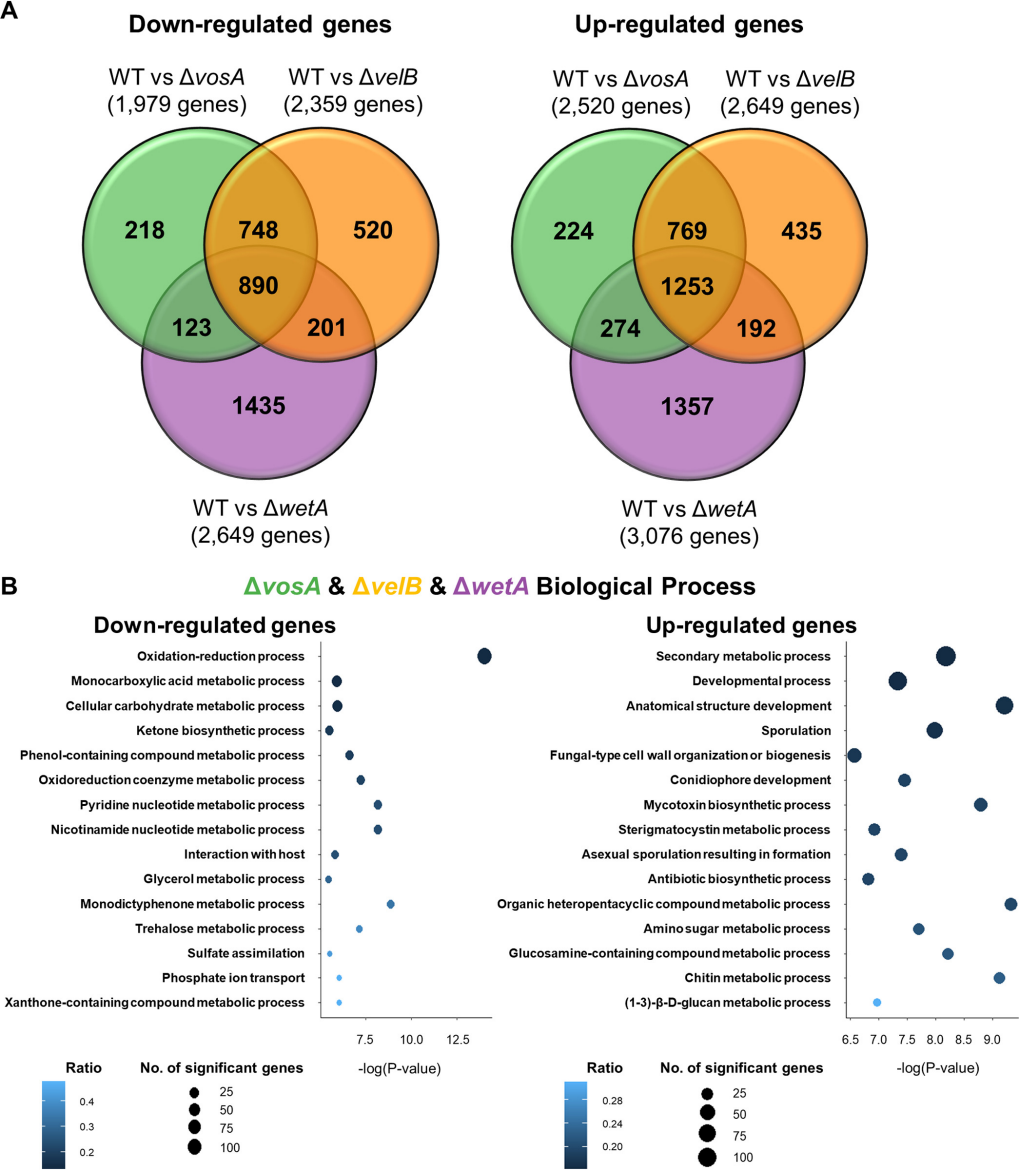
Genome-wide analyses of the genes differentially affected by VosA, VelB, and WetA in A. nidulans conidia. (A) Venn diagram showing the genes whose mRNA levels are downregulated (left) or upregulated (right) by the absence of VosA, VelB, or WetA in conidia. (B) Gene Ontology (GO) term enrichment analysis of downregulated (left) or upregulated (right) genes in the Δ*vosA*, Δ*velB*, and Δ*wetA* conidia.

To understand the conserved and divergent regulatory roles of VosA, VelB, and WetA *in* A. nidulans conidia, a comparative analysis of gene expression differences between the WT and null mutant conidia was carried out (Fig. 1). Totals of 40.98% (4,503/10,988), 45.61% (5,012/10,988), and 51.96% (5,729/10,988) of genes of the A. nidulans genome are differentially regulated in the Δ*vosA*, Δ*velB*, and Δ*wetA* mutant conidia, respectively, suggesting that the three regulators have a broad regulatory effect on conidia (see Fig. S1 in the supplemental material). A total of 2,143 differentially expressed genes (DEGs) between the WT and the Δ*vosA*, Δ*velB*, and Δ*wetA* mutant conidia were identified (Fig. 1A) (fold change of >2.0 for upregulation or downregulation and *q* value [false discovery rate {FDR}] of <0.05). The mRNA expression levels of 890 genes were downregulated in all three mutant conidia compared with the WT conidia. However, in all three mutant conidia, the mRNA levels of 1,253 genes were upregulated. Among them, the mRNA expression levels of a variety of genes associated with asexual development and signal transduction were affected by these three TFs (Tables S1 and S2). Certain developmental regulator genes such as *abaA*, *brlA*, *flbC*, *nosA*, *nsdC*, *velC*, *vapA*, and *esdC* were upregulated in all three null mutants (Table S1). Genes associated with heterotrimeric G-protein signal transduction (GprC, GprC, GprK, GprM, FlbA, and RgsB) and the mitogen-activated protein (MAP) kinase pathway (MpkB and ImeB) were also upregulated by these TFs in conidia (Table S2). However, several genes related to sporulation, spore wall formation, and structural integrity, including *rodA*, *conJ*, *tpsA*, *wA*, *vadA*, and *atfB*, were downregulated in all three null mutants (Table S1). Importantly, 748 and 769 DEGs were down- or upregulated by both Δ*vosA* and Δ*velB* mutant conidia, respectively, but not Δ*wetA* mutant conidia, while the mRNA levels of 2,792 genes were affected solely in the *wetA*-null mutant conidia. Put together, these results suggest that VosA and VelB share more DEGs, while the WetA regulon has many more uniquely regulated genes.

10.1128/mBio.03128-20.1 FIG S1 Summary of DEGs in the Δ*vosA*, Δ*velB*, and Δ*wetA* conidia. Download FIG S1, TIF file, 0.5 MB. Copyright © 2021 Wu et al. 2021 Wu et al. This content is distributed under the terms of the Creative Commons Attribution 4.0 International license.

10.1128/mBio.03128-20.4 TABLE S1 DEGs related to asexual development in the null mutants’ conidia. Download Table S1, XLSX file, 0.02 MB. Copyright © 2021 Wu et al. 2021 Wu et al. This content is distributed under the terms of the Creative Commons Attribution 4.0 International license.

10.1128/mBio.03128-20.5 TABLE S2 DEGs related to signal transduction in the null mutants’ conidia. Download Table S2, XLSX file, 0.02 MB. Copyright © 2021 Wu et al. 2021 Wu et al. This content is distributed under the terms of the Creative Commons Attribution 4.0 International license.

To gain further insight into the regulatory roles of these TFs, functional category analyses using Gene Ontology (GO) terms were carried out (Fig. 1B). The results of the GO analysis demonstrated that several genes involved in the monocarboxylic acid metabolic process, the oxidation-reduction process, the trehalose metabolic process, and the cellular carbohydrate metabolic process were downregulated in all three mutant conidia, whereas a large number of genes associated with the secondary metabolic biosynthetic process, the chitin biosynthetic process, asexual sporulation resulting in formation, and the (1-3)-β-d-glucan metabolic process were upregulated in these mutant conidia. The VosA- and VelB-specific downregulated genes were enriched in functional categories that included the cellular catabolic process, protein localization, and the acetate catabolic process. The functional GO categories associated with the VosA- and VelB-specific upregulated genes were the secondary metabolic biosynthetic process, the steroid metabolic process, and transport (Fig. S2A). Interestingly, a large number of genes involved in the RNA metabolic process were downregulated in Δ*wetA* mutant conidia but not in Δ*vosA* or Δ*velB* mutant conidia (Fig. S2B).

10.1128/mBio.03128-20.2 FIG S2 Gene Ontology (GO) term enrichment analysis of DEGs in the Δ*vosA*, Δ*velB*, and Δ*wetA* conidia. Shown are the top enriched functional categories of the biological process GO terms of DEGs in both Δ*vosA* and Δ*velB* conidia (A) or in Δ*wetA* conidia (B). Download FIG S2, TIF file, 0.5 MB. Copyright © 2021 Wu et al. 2021 Wu et al. This content is distributed under the terms of the Creative Commons Attribution 4.0 International license.

### Putative direct targets of VosA, VelB, and/or WetA in conidia.

**FIG 2:**
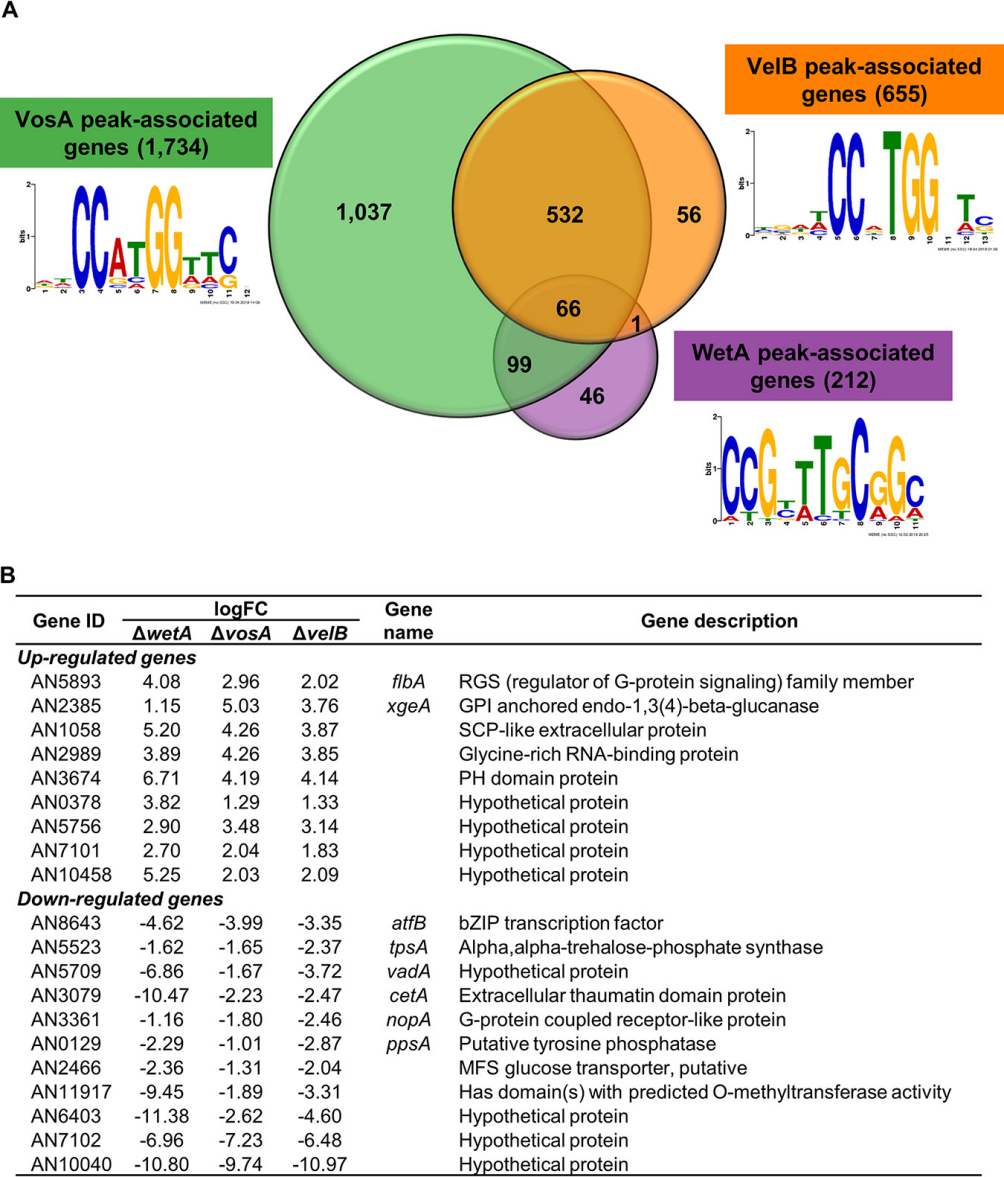
Identification of VosA, VelB, and WetA direct targets in A. nidulans conidia. (A) Venn diagram showing the number of the VosA, VelB, and WetA peak-associated genes in conidia. Motifs identified in peak-associated genes are shown next to the labels. (B) Summary of potential VosA, VelB, and WetA direct target DEGs in A. nidulans conidia. GPI, glycosylphosphatidylinositol; SCP, sperm-coating protein; PH, pleckstrin homology; bZIP, basic leucine zipper; MFS, major facilitator superfamily.

Our previous studies reported that VosA contains the velvet DNA-binding domain, which recognizes the VosA-binding motif in certain promoter regions (29). To identify the VelB direct target genes and compare the putative direct target genes of VosA and VelB, ChIP experiments followed by high-throughput sequencing of the enriched DNA fragments were carried out. ChIPs from strains containing FLAG epitope-tagged versions of VosA and VelB were compared to ChIPs from WT conidia that did not contain the FLAG epitope. Totals of 1,734 and 655 genes that were VosA and VelB peak associated, respectively, were identified using the same analysis pipeline as the one described previously (25) (Fig. 2). To identify the VosA/VelB response elements, DNA sequences in the 100 bp surrounding each peak were subjected to Multiple Em for Motif Elicitation (MEME) analysis, which led to the predicted VosA response element (VoRE) and the predicted VelB response element (VbRE) (Fig. 2A). Interestingly, the predicted VbRE (5′-CCXTGG-3′) was quite similar to the predicted VoRE (5′-CCXXGG-3′). The VoRE was found in 278/1,404 peak sequences, had an E value of 1.6e−56, and was the only motif identified by MEME with an E value of <1. The VbRE was found in 188/511 peak sequences, had an E value of 2.4e−85, and was one of only two motifs identified by MEME with an E value of <1 (the other motif had an E value of 4.0e−5 and was found in only 72 peak sequences).

We then compared the results of the ChIP-seq and RNA sequencing (RNA-seq) analyses to identify potential direct target genes of the three TFs (Table S3). There were 66 genes associated with the peaks of all three TFs (Table S4). Among them, 22 genes, including *flbA*, *xgeA*, *atfB*, *tpsA*, *vadA*, *cetA*, *nopA*, and *ppsA*, were DEGs in all three null mutants (Fig. 2B). Importantly, 532 genes were considered to be potential direct target genes for both VosA and VelB but not WetA. A total of 166 genes were upregulated in both Δ*vosA* and Δ*velB* mutant conidia. These genes, including *brlA*, *fadA*, *rosA*, *steA*, *steC*, and *veA*, were found to be involved primarily in asexual or sexual developmental processes. Taking these results together with the previously reported results (27, 35), we suggest that VosA works with VelB and that the VosA-VelB complex coordinates the processes involved in conidial production and maturation in A. nidulans.

10.1128/mBio.03128-20.6 TABLE S3 VosA, VelB, and WetA peak-associated DEGs. Download Table S3, XLSX file, 0.1 MB. Copyright © 2021 Wu et al. 2021 Wu et al. This content is distributed under the terms of the Creative Commons Attribution 4.0 International license.

10.1128/mBio.03128-20.7 TABLE S4 Sixty-six DEGs associated with VosA, VelB, and WetA peaks. Download Table S4, XLSX file, 0.01 MB. Copyright © 2021 Wu et al. 2021 Wu et al. This content is distributed under the terms of the Creative Commons Attribution 4.0 International license.

### Roles of VosA, VelB, and/or WetA in conidial wall integrity.

**FIG 3:**
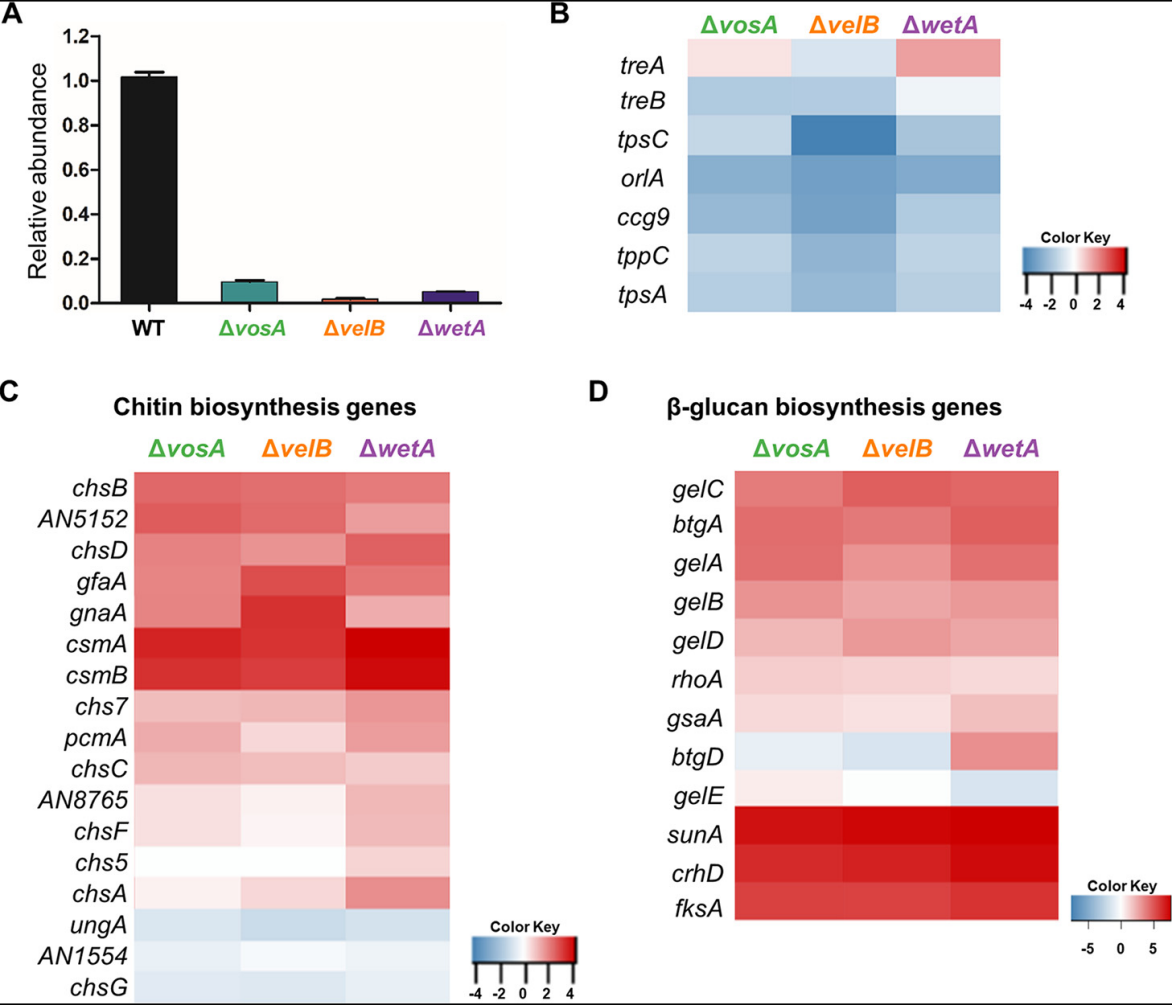
Regulatory effects of VosA, VelB, and WetA on trehalose, chitin, and β-1,3-glucan biosynthesis in A. nidulans conidia. (A) Amount of conidial trehalose in A. nidulans. (B to D) Levels of mRNA of the genes associated with trehalose levels (B), chitin biosynthesis (C), and β-1,3-glucan biosynthesis (D) in the Δ*vosA*, Δ*velB*, and Δ*wetA* conidia. logFC, log fold change; GPI, glycosylphosphatidylinositol; MFS, major facilitator superfamily.

Previous studies have shown that the deletion of *vosA*, *velB*, or *wetA* leads to decreased amounts of trehalose and increased β-glucan levels in conidia (25, 30). The results of transmission electron microscopy analyses revealed that three TFs are needed for the proper formation of the conidial wall (25, 30, 37), suggesting that these genes play a conserved role in regulating the expression of genes associated with conidial structural integrity. High-performance liquid chromatography (HPLC) analysis demonstrated that the trehalose contents of the three null mutant conidia were dramatically decreased (Fig. 3A). The mRNA expression levels of most genes involved in trehalose biosynthesis were downregulated (Fig. 3B and Table S5). In addition, *tpsA*, a putative trehalose synthase gene, is the direct target of three TFs (Fig. 2B). These results suggest that three TFs directly or indirectly control the mRNA levels of genes associated with trehalose biosynthesis, thereby regulating the trehalose contents in conidia. Most genes associated with chitin and β-(1,3)-glucan biosynthesis were upregulated in the Δ*vosA*, Δ*velB*, and Δ*wetA* mutant conidia (Fig. 3C and D). These results suggest that VosA, VelB, and WetA govern the mRNA expression of genes associated with conidial wall integrity in A. nidulans.

10.1128/mBio.03128-20.8 TABLE S5 DEGs involved in conidial wall integrity in the null mutants’ conidia. Download Table S5, XLSX file, 0.02 MB. Copyright © 2021 Wu et al. 2021 Wu et al. This content is distributed under the terms of the Creative Commons Attribution 4.0 International license.

### Alterations to primary metabolites in Δ*vosA*, Δ*velB*, and Δ*wetA* conidia.

**FIG 4:**
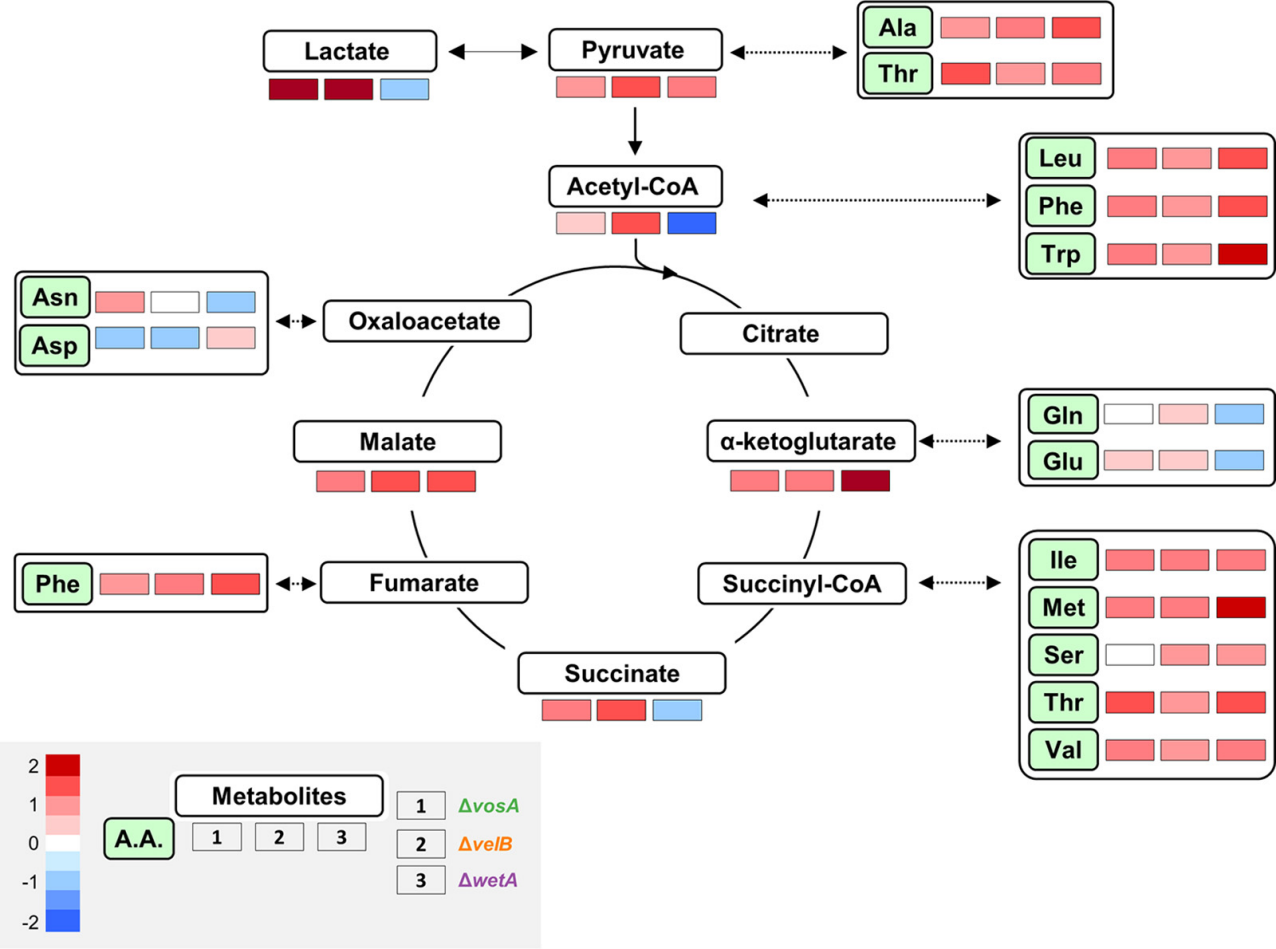
Roles of VosA, VelB, and WetA in primary metabolism of A. nidulans conidia. Shown are maps of primary metabolites involved in the TCA cycle and amino acid (A.A.) biosynthesis in WT, Δ*vosA*, Δ*velB*, and Δ*wetA* conidia. Levels of identified primary metabolites produced in the WT and null mutant conidia are shown.

As mentioned above, the deletion of *vosA*, *velB*, or *wetA* led to alterations in the mRNA expression of genes involved in metabolic processes (glycerol metabolic process, ketone metabolic process, and amino sugar metabolic process) and amino acid metabolism (Table S6), implying that the amounts of primary metabolites may be affected by the absence of *vosA*, *velB*, or *wetA* in conidia. To test this hypothesis, the abundances of several primary metabolites involved in the tricarboxylic acid (TCA) cycle and amino acid biosynthesis were examined in WT and mutant conidia (Fig. 4). The abundances of pyruvate, α-ketoglutarate, and malate were increased in the conidia of the three null mutants. The abundances of acetyl-CoA and succinate were decreased in both Δ*vosA* and Δ*velB*, but not Δ*wetA*, mutant conidia. The amounts of lactate in both Δ*vosA* and Δ*velB* mutant conidia were significantly large compared with those in WT conidia.

10.1128/mBio.03128-20.9 TABLE S6 DEGs involved in amino acid metabolism in the null mutants’ conidia. Download Table S6, XLSX file, 0.02 MB. Copyright © 2021 Wu et al. 2021 Wu et al. This content is distributed under the terms of the Creative Commons Attribution 4.0 International license.

The abundances of 13 amino acids (alanine, isoleucine, methionine, leucine, phenylalanine, tryptophan, valine, threonine, serine, asparagine, glutamine, aspartate, and glutamate) were affected in at least one null mutant. Moreover, the levels of nine amino acids were high in all three mutant conidia. The effects of deleting *vosA*-*velB* or *wetA* on the abundances of glutamate, glutamine, aspartate, and asparagine differed. The deletion of *wetA* caused decreased levels of glutamate, glutamine, and asparagine in conidia, whereas the levels of these amino acids were increased or not affected by the absence of *vosA* or *velB*. The genes involved in the biosynthesis of these amino acids and primary metabolites were differentially regulated in the three null mutants. Overall, these results show that WetA, VosA, and VelB regulate the expression of genes involved in both the TCA cycle and amino acid biosynthesis; however, the three lists of primary metabolites that they affect contain both shared and unique molecules.

### Abundances of secondary metabolites in Δ*vosA*, Δ*velB*, and Δ*wetA* conidia.

Previous studies found that these three TFs are important for the production of several secondary metabolites in *Aspergillus* species (34, 36, 40). In addition, according to the GO analysis results, the deletion of *vosA*, *velB*, or *wetA* results in an alteration of the mRNA expression of biosynthetic gene clusters involved in the production of multiple secondary metabolites, including monodictyphenone, sterigmatocystin, and asperfuranone (Fig. 1, Fig. S2, and Table S7). To elucidate the conserved and divergent regulatory effects of secondary metabolism in the three conidial mutants, the secondary metabolites were extracted and subjected to liquid chromatography-mass spectrometry (LC-MS) analysis. A principal-component analysis showed differences between the four different conidial samples (Fig. S3). The secondary metabolite content of the WT conidia was relatively similar to that of the Δ*wetA* conidia, indicating similar abundances and types of secondary metabolites. Conidia from the Δ*vosA* and Δ*velB* mutants clustered far apart, which suggested that a unique set of secondary metabolites or different levels of metabolites were expressed and extracted. This is interesting considering that the two TFs can interact and that their binding motifs and regulated gene lists were so similar to one another (Fig. 1A and Fig. 2A).

10.1128/mBio.03128-20.3 FIG S3 Principal-component analysis of the metabolic differences between the conidial metabolites from the WT, Δ*vosA*, Δ*velB*, and Δ*wetA* strains. Download FIG S3, TIF file, 0.3 MB. Copyright © 2021 Wu et al. 2021 Wu et al. This content is distributed under the terms of the Creative Commons Attribution 4.0 International license.

10.1128/mBio.03128-20.10 TABLE S7 DEGs contained in the secondary metabolism gene clusters in the null mutants’ conidia. Download Table S7, XLSX file, 0.04 MB. Copyright © 2021 Wu et al. 2021 Wu et al. This content is distributed under the terms of the Creative Commons Attribution 4.0 International license.

**FIG 5:**
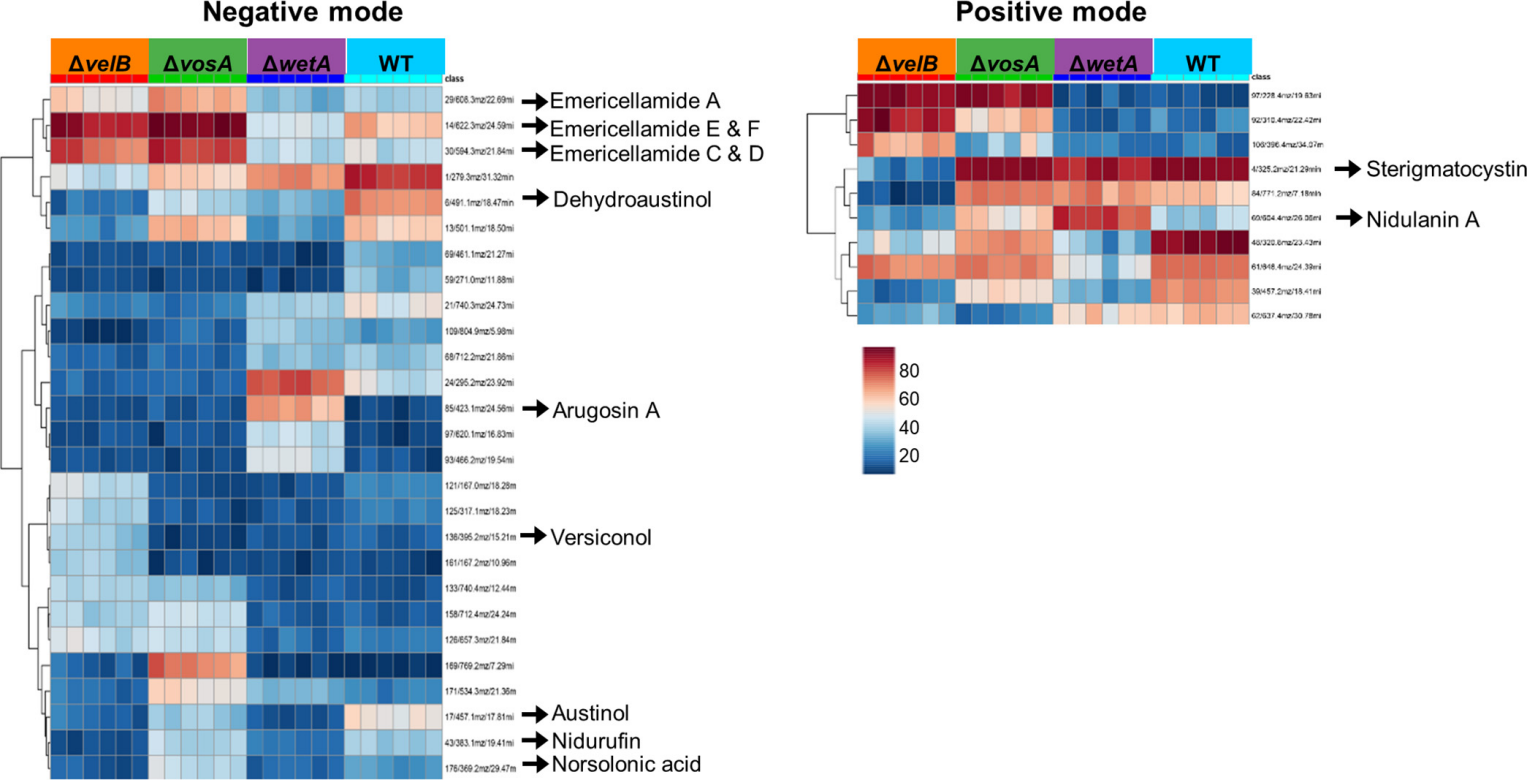
Levels of secondary metabolites in the Δ*vosA*, Δ*velB*, and Δ*wetA* conidia. Differentially regulated secondary metabolites in WT, Δ*vosA*, Δ*velB*, and Δ*wetA* conidia are shown. The heat map is color-coded and represents high abundances (red) or low abundances (blue) of ions/retention time pairs detected by LC-MS analysis.

Next, we applied analysis of variance to identify the most different molecular entities detected as mass/charge (*m/z*) values and retention time (RT) pairs in the LC-MS analysis-derived metabolomics data. As shown in Fig. 5, the abundances of several secondary metabolites were different in the positive and negative ionization modes. For example, the abundance of arugosin A was high in the Δ*wetA* conidia, compared with the WT conidia, but not in the Δ*vosA* and Δ*velB* mutant conidia.

**FIG 6:**
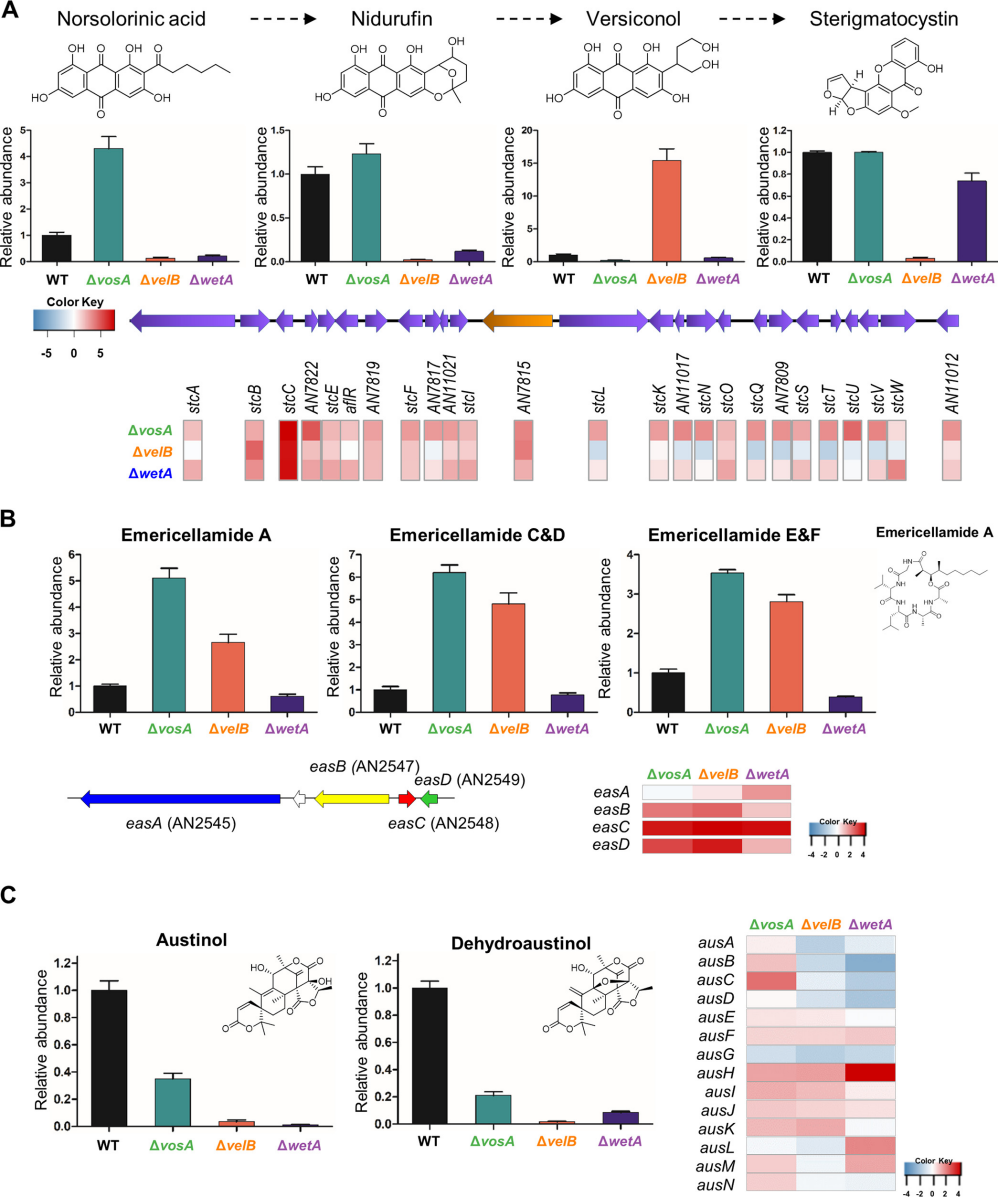
Regulation of key secondary metabolites in Δ*vosA*, Δ*velB*, and Δ*wetA* conidia of A. nidulans. (A, top) Chemical structures of the compounds. (Middle) Abundances of norsolorinic acid, nidurufin, versiconol, and sterigmatocystin in WT, Δ*vosA*, Δ*velB*, and Δ*wetA* conidia. (Bottom) The sterigmatocystin gene cluster and differentially expressed genes involved in sterigmatocystin biosynthesis in Δ*vosA*, Δ*velB*, and Δ*wetA* conidia. (B, top) Abundance of emericellamide in WT, Δ*vosA*, Δ*velB*, and Δ*wetA* conidia with the emericellamide A structure. (Bottom) The emericellamide gene cluster and mRNA expression of genes associated with emericellamide biosynthesis in Δ*vosA*, Δ*velB*, and Δ*wetA* conidia. (C, left) Abundances of austinol and dehydroaustinol in WT, Δ*vosA*, Δ*velB*, and Δ*wetA* conidia with their structures. (Right) The austinol gene cluster and mRNA expression of genes associated with austinol biosynthesis in Δ*vosA*, Δ*velB*, and Δ*wetA* conidia.

To further dissect the roles of VosA, VelB, and WetA in secondary metabolism, we focused on some known secondary metabolites, including sterigmatocystin, emericellamide, and austinol (Fig. 6). Sterigmatocystin is a precursor of aflatoxins, and its biosynthetic gene cluster and intermediates have previously been studied (41, 42). The amount of sterigmatocystin in the Δ*velB* conidia was significantly decreased compared with that in the WT conidia, but the Δ*vosA* and Δ*wetA* conidia contained similar amounts of sterigmatocystin (Fig. 6A). However, the amounts of sterigmatocystin intermediates were different in Δ*vosA* and Δ*wetA* conidia. Levels of norsolorinic acid and nidurufin were low in the Δ*velB* and Δ*wetA* conidia, while the level of versiconol was high only in the Δ*velB* conidia. The RNA-seq results indicated that the mRNA levels of almost all of the genes in the sterigmatocystin gene cluster were increased in both the Δ*vosA* and Δ*wetA* conidia, whereas the mRNA expression of these genes in the Δ*velB* conidia was less consistent. In particular, the mRNA levels of *stcL*, *stcN*, *stcQ*, *stcS*, *stcT*, *stcU*, *stcV*, and *stcW* were decreased in the Δ*velB* conidia compared with the WT conidia. These results suggest that VosA and VelB play diverse roles in the regulation of sterigmatocystin biosynthesis.

Emericellamide compounds are cyclopeptides that are produced by several *Aspergillus* species (43, 44). The abundances of these compounds, relative to WT production, were high in Δ*vosA* and Δ*velB* conidia, and the mRNA levels of *easA, easB, easC,* and *easD* were also high in both mutant conidia, implying that VosA and VelB repress emericellamide biosynthesis in WT conidia (Fig. 6B). In the Δ*wetA* conidia, however, the mRNA expression of the emericellamide gene cluster was increased, but the quantity of emericellamide compounds did not increase, suggesting that the regulatory mechanism of emericellamide biosynthesis in the Δ*wetA* conidia is more complex than the influence of Δ*vosA* and Δ*velB* on emericellamide production in conidia. In the three types of null mutant conidia, the abundances of two fungal meroterpenoids, austinol and dehydroaustinol (45), were decreased, compared with the WT conidia (Fig. 6C). Furthermore, the expression levels of several austinol cluster genes were decreased in the Δ*velB* and Δ*wetA* conidia. Taken together, these results demonstrate that the ways in which VosA, VelB, and WetA govern the expression of secondary metabolite gene clusters, and the production of their associated metabolites, in A. nidulans conidia are divergent from one another.

## DISCUSSION

Asexual developmental processes in filamentous fungi are regulated by a variety of TFs (6). These TFs orchestrate the spatial and temporal transcriptional expression of development-specific genes, leading to physiological and metabolic changes. During the processes of conidium formation from phialides and conidial maturation, conidium-specific TFs, including VosA, VelB, and WetA, regulate spore-specific gene expression patterns and metabolic changes (25, 30). In this study, we investigated the transcript and metabolite changes that are regulated by VosA, VelB, and WetA in A. nidulans conidia.

Transcriptomic analyses indicated that about 20% of the A. nidulans genome (2,143 genes) is differentially expressed in Δ*vosA*, Δ*velB*, and Δ*wetA* mutant conidia. ChIP-seq results identified 66 direct target genes that are shared between VosA, VelB, and WetA in conidia. These results offered some explanation of how these TFs control phenotypic changes in conidia. First, the deletion of *vosA*, *velB*, or *wetA* caused increased mRNA expression of certain development-specific genes, including *abaA* (23), *brlA* (19), *flbA* (46), *flbC* (47), *nsdC* (48), *nosA* (49), and *mpkB* (50), which are involved in the formation of asexual and sexual structures during the early and middle stages of conidium formation, but decreased transcript accumulation of spore-specific genes such as *vadA* (51), *catA* (52), *wA* (53), *conF* (54), *conJ* (54), *cetA* (55), *cetJ* (56), and *cetL* (56), which are important for conidial germination, morphogenesis, and dormancy (see Table S1 in the supplemental material). Alteration of the mRNA expression levels of development-specific genes or spore-specific genes affect spore maturation, dormancy, and germination. For example, misscheduled expression of key asexual developmental regulators, especially BrlA and AbaA, can affect proper sporulation (9, 57). In the case of the spore-specific genes, the deletion of *vadA* or *catA* affects conidial germination and the conidial stress response (51, 52, 58). Based on these results, we propose that alteration of the mRNA expression levels of development-specific genes or spore-specific genes caused by the deletion of *vosA*, *velB*, or *wetA* affect conidial maturation, dormancy, morphology, and germination. However, the detailed molecular mechanism of how three TFs act as activators or repressors for the expression of development-specific genes and spore-specific genes will be elucidated in further studies.

Another important phenotype of the Δ*vosA*, Δ*velB*, and Δ*wetA* mutant conidia was the differences in conidial wall integrity and the components of the conidial wall (25, 30). As shown in Fig. 3, most of the genes involved in chitin and β-glucan biosynthesis were upregulated in all three mutant conidia. The dynamic expression of these genes is required mainly for the remodeling of the cell wall during isotropic growth and mobilization of energy for differentiation (59) but is not required in dormant conidia. However, by altering the mRNA expression of these genes in the mutant conidia, the dormancy of conidia could be broken, affecting long-term viability as well as conidial germination.

Another feature of fungal spores is their ability to resist various environmental stresses (1). However, Δ*vosA*, Δ*velB*, and Δ*wetA* mutant conidia are more sensitive to several environmental stresses (25, 35). It is speculated that this is regulated by alterations in the expression of genes involved in environmental stress tolerance. The data that we show here support this hypothesis. First, these regulators govern the mRNA expression of genes involved in the trehalose biosynthetic pathway, thereby affecting the amount of conidial trehalose, a key component in stress protection and fungal virulence (60). Second, VosA, VelB, and WetA directly or indirectly regulate genes previously associated with stress responses. CatA is a spore-specific catalase, and compared with WT spores, *catA* deletion mutant spores are sensitive to oxidative stress (52). AtfB is a bZIP TF (61), and the AtfB homolog is crucial for the stress response in Aspergillus oryzae conidia (62). These two genes are putative direct target genes of the three regulators reported in this study, and the mRNAs of *catA* and *atfB* can be positively regulated by VosA, VelB, and WetA in conidia (Fig. 2 and Table S3). Along with these genes, the mRNA level of *hogA*, a key component of osmotic stress signaling (63), was downregulated in all mutant conidia. These results contribute to our understanding of the ways in which these three regulators influence the environmental stress response in conidia.

VosA, VelB, and WetA are key functional regulators in the formation of conidia and control spore-specific gene expression. However, our data have shown that their gene regulation networks are slightly different. RNA-seq results showed that VosA and VelB coregulate the expression of spore-specific genes. Importantly, the predicted VbRE is quite similar to the predicted VoRE (Fig. 2A). In addition, biochemical results from previous studies (27, 35) suggested that VosA and VelB form a heterocomplex in asexual spores. However, WetA is not directly related to VosA and VelB. WetA’s putative binding site is different from the VosA/VelB binding site. Moreover, the WetA peak-associated genes and the VosA/VelB peak-associated genes did not overlap much. These results imply that WetA-mediated gene regulation may be different from the VosA- or VelB-mediated gene regulatory network.

The velvet domain is a fungus-specific DNA-binding domain that recognizes specific DNA sequences. Previously, Ahmed et al. proposed that the VosA velvet domain recognizes a DNA sequence (5′-TGGCCGCGG-3′) based on ChIP-chip analysis and electrophoretic mobility shift assays (EMSAs) (29). Further EMSAs demonstrated that both TGG and CCGCGG sequences are necessary for DNA binding of the VosA velvet domain. In the present study, we conducted ChIP-seq analyses in conidia and proposed the predicted VbRE (5′-CCXTGG-3′) and VoRE (5′-CCXXGG-3′) (Fig. 2). In our experimental results, the TGG sequence does not appear for the VbRE or VoRE, but the 5′-CCXXGG-3′ sequence is conserved in the VbRE and VoRE. The reason why these DNA sequences are not the same is likely because the experimental methods and analyses are different from those used to obtain the previous results. Ahmed et al. used 15 DNA sequences based on chromatin immunoprecipitation with microarray technology (ChIP-chip) analysis and EMSAs, whereas the motif in Fig. 2A was built from running MEME with every peak sequence that we identified. Nevertheless, the 5′-CCXXGG-3′ sequence appears common in previous and current results. Based on these data, we propose that the 5′-CCXXGG-3′ sequence may be crucial for DNA binding of the velvet domain, and further studies will be needed to fine-tune the precise velvet protein-binding sequence.

During the asexual development of A. nidulans, the abundance of amino acids other than phenylalanine changes, and the expression of genes related to amino acid biosynthesis is altered (64). Overall, our analyses confirmed that the amounts of most amino acids, and the expression of related genes, increased in all mutant spores. In addition, the abundances of metabolites involved in the TCA cycle increased in all mutant conidia. However, the abundances of some primary metabolites such as glutamate, glutamic acid, lactate, and acetyl-CoA were decreased in the Δ*wetA* conidia (Fig. 4). It is not yet clear how these metabolic changes affect spore production and maturation, and further studies will be needed to understand this.

Our multi-omics analyses found that VosA, VelB, and WetA regulate the expression of several secondary metabolite gene clusters (Table S7) and the production of secondary metabolites, especially sterigmatocystin, in conidia. The process of sterigmatocystin production and its regulation involves 25 genes, and this metabolite is produced via steps involving several intermediate products. In Δ*vosA* conidia, the mRNA expression of sterigmatocystin gene clusters was induced, and the amounts of sterigmatocystin produced were similar to those in the WT conidia. These results were similarly observed in sexual spores (34). While the Δ*vosA* conidia contained sterigmatocystin, the metabolite was not detected in Δ*velB* conidia. We reported that the VosA-VelB complex is a functional unit in conidia, but this particular result indicates that VosA and VelB play different roles in sterigmatocystin production. It is possible that VelB forms another complex, such as the VelB-VeA-LaeA complex (40), to participate in sterigmatocystin production in conidia. For the Δ*velB* conidia, we speculated that the mRNA expression levels of genes such as *stcB*, *stcC*, *stcF*, and *stcI*, which are associated with the early stages of sterigmatocystin biosynthesis, were increased, and that the amount of versiconol, a putative sterigmatocystin/aflatoxin intermediate, was also increased in comparison with the WT. However, the mRNA levels of genes associated with the late phase of sterigmatocystin biosynthesis, such as *stcL*, *stcN*, *stcQ*, and *stcT*, were decreased in Δ*velB* conidia. It might be possible that VelB (or VelB/VeA/LaeA) can regulate some expression of sterigmatocystin gene clusters by epigenetic means rather than through the canonical method of *aflR* expression or activity. Although changes in the expression of secondary metabolite gene clusters and secondary metabolites affected by three TFs were studies, detailed molecular mechanisms have not been studied yet. Therefore, it is necessary to study how these three TFs work together or separately through further research. In the Δ*vosA* and Δ*wetA* conidia, the mRNA levels of most of the genes in the sterigmatocystin gene cluster were increased compared to those in WT conidia, but the amounts of sterigmatocystin were similar to those in WT conidia. There are some speculations about this phenomenon. The expression of genes may not directly affect the biosynthesis of secondary metabolites. Alternatively, the translation of mRNA molecules to proteins and the posttranslational modification of those metabolite-producing proteins are two factors that can create discrepancies between RNA and metabolite abundances. To further explain this, further experiments should be conducted to determine how the three TFs regulate the biosynthesis of secondary metabolites.

**FIG 7:**
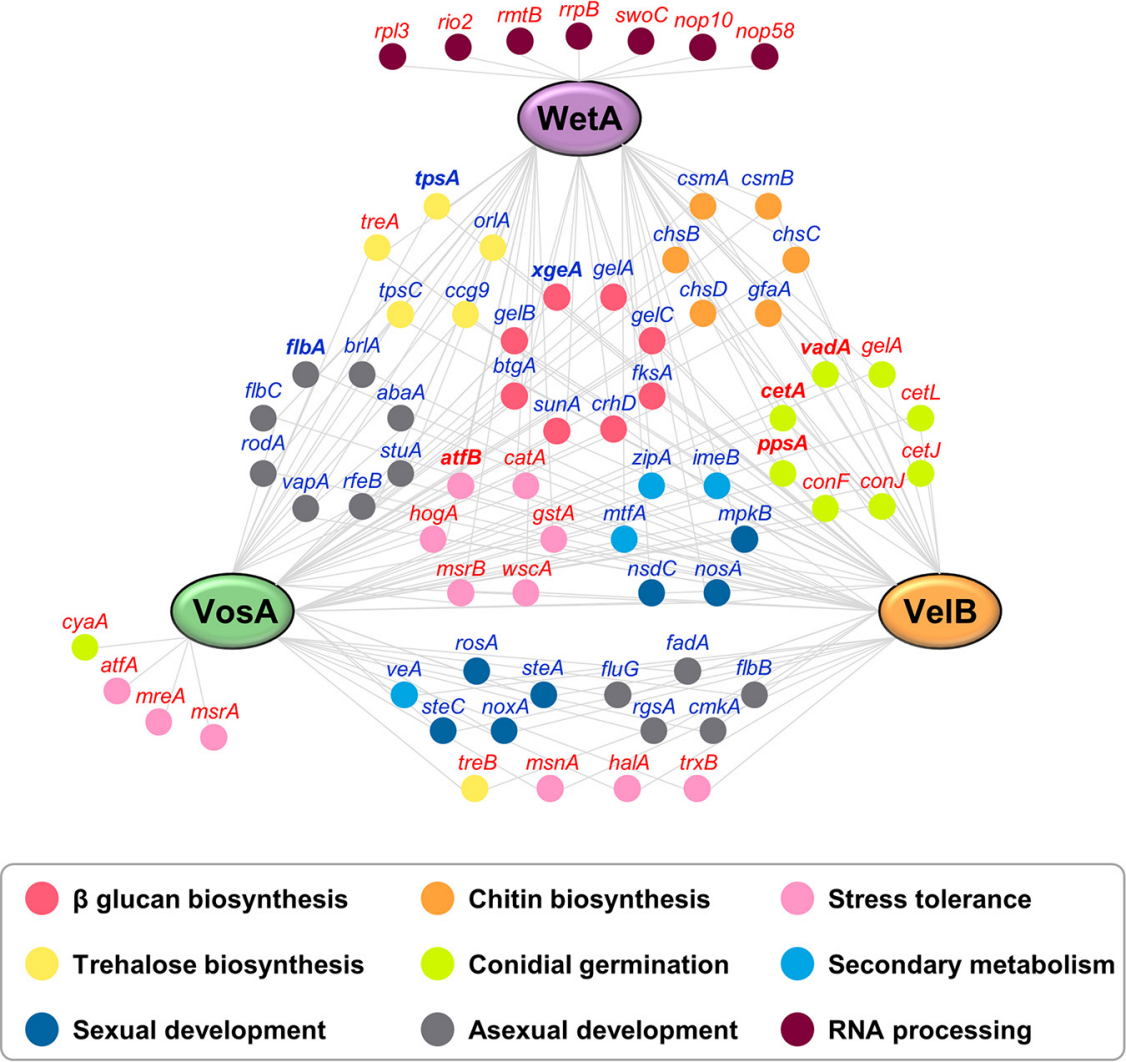
Proposed gene regulatory network of VosA, VelB, and WetA in conidia. The network represents the interactions between VosA/VelB/WetA and their target genes. Gene names in boldface type are direct target genes of all three TFs. Gene names in red or blue are genes induced or repressed, respectively, by VosA, VelB, and/or WetA in conidia.

In conclusion, this study provides a systematic dissection of the gene regulatory network and molecular mechanisms of VosA, VelB, and WetA (Fig. 7). In conidia, VosA, VelB, and WetA directly or indirectly control the expression of spore-specific or development-specific genes, thereby altering conidial wall integrity and conidial viability. In addition, these TFs regulate multiple secondary metabolite gene clusters, thus inducing secondary metabolic changes. These results provide an advance in the knowledge of conidial formation and will provide the basis for future insights into spore formation in other filamentous fungi.

## MATERIALS AND METHODS

### Strains, media, and culture conditions.

The fungal strains used in this study are listed in Table 1. Fungal strains were grown on solid or liquid minimal medium with 1% glucose (MMG) and appropriate supplements for general purposes as previously described (65). For conidium samples, WT and mutant conidia were inoculated onto solid MMG plates and incubated for 48 h. Next, conidia were collected from plates using Miracloth (Calbiochem, San Diego, CA, USA) and stored at −80°C.

**TABLE 1:** *Aspergillus* strains used in this study

Strain	Relevant genotype	Source or reference
FGSC4	A. nidulans wild type; *veA^+^*	FGSC^*a*^
THS15	*pyrG89*; *pyroA4*; Δ*vosA*::Afu*pyrG*^+^; *veA*^+^	27
THS16	*pyrG89*; *pyroA4*; Δ*velB*::Afu*pyrG*^+^; *veA*^+^	27
THS20.1	*pyrG89*; *pyroA*::*velB*(p)::*velB*::FLAG_3×_::*pyroA*^*b*^; Δ*velB*::Afu*pyrG*^+^; *veA*^+^	27
THS28.1	*pyrG89*; *pyroA*::*vosA*(p)::*vosA*::FLAG_3×_::*pyroA*^*b*^; Δ*vosA*::Afu*pyrG*^+^; *veA*^+^	27
TMY4	*pyrG89*; *pyroA4*; Δ*wetA*::Afu*pyrG*^+^; *veA*^+^	25

a FGSC, Fungal Genetic Stock Center.

b The 3/4 *pyroA* marker causes targeted integration at the *pyroA* locus.

### RNA sequencing analysis.

To isolate total RNA for RNA sequencing (RNA-seq) analysis, total RNA from WT and mutant conidia was extracted using TRIzol reagent (Invitrogen, USA), according to the manufacturer’s instructions, with modifications. To remove DNA contamination from the RNA samples, DNase I (Promega, USA) was added, and RNA was then purified using an RNeasy minikit (Qiagen, USA). Three technical replicates of each sample were analyzed. RNA sequencing was performed as previously described (34). RNA samples were submitted to the University of Wisconsin Gene Expression Center (Madison, WI, USA) for library preparation and sequencing. A strand-specific library was prepared using an Illumina TruSeq strand-specific RNA sample preparation system. The libraries of all the replicates were sequenced using an Illumina HiSeq 2500 system.

Data analysis of the Δ*vosA* and Δ*velB* RNA-seq experiments was performed using the same analysis pipeline as the one previously described for the Δ*wetA* RNA-seq analysis (25). Reads were mapped to the A. nidulans FGSC4 transcriptome using Tophat2 version 2.1.1 (66) and the parameter “–max-intron-length 4000.” On average, 19.9 million reads per sample mapped to the genome, and the number of reads aligning to each gene was counted with HTseq-Count version 0.9.1 (67). DESeq version 1.14.1 (68) was used to determine significantly differentially expressed genes, and genes were considered regulated if they exhibited an adjusted *P* value of <0.05 and a log_2_ fold change either greater than 1 or less than −1.

### Chromatin immunoprecipitation sequencing analysis.

Samples for chromatin immunoprecipitation sequencing (ChIP-seq) analysis were prepared according to methods described previously (29, 30). DNA samples from each strain were extracted using a MAGnify chromatin immunoprecipitation system (Invitrogen, USA) according to the manufacturer’s protocol, with modification. Two-day-old conidia from the WT strain or strains containing VosA-FLAG or VelB-FLAG were cross-linked, washed, homogenized with a Mini-Beadbeater 16 instrument (Biospec, USA), sonicated, and separated by centrifugation. The chromatin extracts were incubated with an anti-FLAG antibody–Dynabead complex. Next, samples were eluted from the beads at 55°C using proteinase K. Enriched DNA was purified using DNA purification magnetic beads. DNA samples from each strain were submitted to the University of Wisconsin Gene Expression Center (Madison, WI). Libraries were prepared using a TruSeq ChIP library preparation kit (Illumina, CA). The libraries of all the replicates were sequenced using an Illumina HiSeq 2500 system.

Raw reads were trimmed using Trimmomatic version 0.36 (69) and the parameters “ILLUMINACLIP:2:30:10 LEADING:3 TRAILING:3 SLIDINGWINDOW:4:15 MINLEN:36.” Trimmed reads were mapped to the A. nidulans A4 genome using version 0.7.15 of BWA-MEM (70), and shorter split hits were marked as secondary alignments. Mapped reads with mapping quality (MAPQ) values of <1 as well as unmapped, secondarily aligned, supplementary, and duplicated reads were discarded with SAMtools version 1.6 (71). On average, 2.3 million and 7.2 million reads per sample were used for peak calling in the VosA and VelB experiments, respectively. Mapped reads that survived our filter were pooled, and extension sizes were estimated with version 1.15.2 of SPP (72, 73). Peaks were called with MACS2 (74) version 2.1.2 using the extension sizes estimated by SPP, a genome size of 2.93e7, and the “–nomodel” parameter. Peaks with a fold change of >2.0 and a *q* value of <0.001 were further analyzed. Peak lists were combined from both of the VosA biological replicates, as >99% of the peaks from the first replicate were found in the second replicate. Motifs were identified in the 100 bp of sequences surrounding each peak summit using MEME-ChIP (75). Motifs that occurred zero times or once in the sequences around the peaks and that were 4 to 21 nucleotides (nt) long were further analyzed.

### Functional enrichment analysis.

Enriched terms from the GO Biological Process, KEGG, InterPro, and Pfam databases were identified using the tools available at AspGD (76), FungiDB (77), and ShinyGO v0.60 (78). Unless otherwise stated, default settings were used in ShinyGO v0.60. The settings were as follows: database, *Emericella nidulans* STRINGdb; *P* value cutoff (FDR), 0.05; number of most significant terms to show, 30.

### Primary metabolite analysis.

WT and Δ*wetA*, Δ*vosA*, and Δ*velB* mutant conidia were inoculated onto solid MMG plates and incubated for 48 h, and fresh conidia were then harvested using Miracloth with HPLC-grade water. For each sample, 2 × 10^8^ conidia were mixed with 500 μl HPLC-grade acetonitrile-methanol-water (40:40:20, vol/vol) and 300 μl beads, homogenized by using the Mini-Beadbeater, and centrifuged. The supernatant was filtered using a 0.45-μm polytetrafluoroethylene (PTFE) Mini-UniPrep filter vial (Agilent), collected, and immediately snap-frozen with liquid nitrogen. The samples were stored at −80°C until primary metabolite analysis.

The samples were then analyzed as described previously (79, 80). Samples were analyzed using an HPLC-MS system consisting of a Dionex ultrahigh-performance liquid chromatography (UHPLC) instrument coupled by electrospray ionization (ESI) (negative mode) to a hybrid quadrupole–high-resolution mass spectrometer (Q Exactive orbitrap; Thermo Scientific) operated in full-scan mode. Metabolite peaks were identified by their exact mass and matching retention times to those of pure standards (Sigma-Aldrich).

### Secondary metabolite analysis.

The conidia of WT and Δ*wetA*, Δ*vosA*, and Δ*velB* mutant strains were extracted by adding 1.5 ml of a methanol-acetonitrile (2:1) mixture followed by sonication for 60 min. The suspension was then left overnight before centrifugation at 14,000 rpm for 15 min. The supernatant (1 ml) was removed, filtered, and evaporated to dryness *in vacuo*. Extracts for the metabolomics analysis were normalized to 10 mg/ml in methanol for LC-MS analysis.

Analytical HPLC was performed using an Agilent 1100 HPLC system equipped with a photodiode array detector. The mobile phase consisted of ultrapure water (mobile phase A) and acetonitrile (mobile phase B) with 0.05% formic acid in each solvent. A gradient method from 10% mobile phase B to 100% mobile phase B in 35 min at a flow rate of 0.8 ml/min was used. The column (Phenomenex Kinetex C_18_, 5 μm by 150 mm by 4.6 mm) was reequilibrated before each injection, and the column compartment was maintained at 30°C throughout each run. All samples were filtered through a 0.45-μm nylon filter before LC-MS analysis.

Extracts from the WT and mutant conidia were analyzed in duplicate on an Agilent 1100 series LC-MS platform (81, 82). The negative ionization mode was found to detect the most metabolites. The first 5 min of every run was removed due to a large amount of coeluting, low-molecular-weight, polar metabolites. Data sets were exported from Agilent’s Chemstation software as .netCDF files and imported into MZmine 2.38 (83). Peak picking was performed with established protocols (84), resulting in 123 marker ions. Briefly, mass detection was centroid with a 5e2 minimum height. Chromatogram building was limited to peaks greater than 0.1 min with a tolerance of 0.05 *m/z* and a minimum height of 1e3. Data smoothing was performed at a filter width of 5. Chromatogram deconvolution was performed by utilizing a local minimum search with a chromatographic threshold of 95%, a minimum relative height of 10%, a minimum absolute height of 3e3, a minimum ratio of peak to edge of 1, and a peak duration range of 0.1 to 5.0 min. The spectra were de-isotoped with a 1-ppm *m/z* tolerance before all treatments were aligned, and duplicate peaks were combined with a tolerance of 0.1 *m/z* and a 3.0-min RT. Peak finder gap filling was performed with 50% intensity tolerance and 0.1 *m/z* tolerance. Peak lists were exported to Metaboanalyst (85), where missing values were replaced with half the minimum positive value, data were filtered by the interquartile range, and log transformation of the data was employed.

### Data availability.

All RNA-seq and ChIP-seq data files are available from the NCBI Gene Expression Omnibus database (*wetA* RNA-seq, accession number GSE114143; *vosA* and *velB* RNA-seq, accession number GSE154639; WetA ChIP-seq, accession number GSE114141; VosA and VelB ChIP-seq, accession number GSE154630).
