# Supplementary figures and images for: Transcriptomic, Protein-DNA Interaction, and Metabolomic Studies of VosA, VelB, and WetA in Aspergillus nidulans Asexual Spores
Source: mBio. 2021 Feb 9;12(1):e03128-20. doi: 10.1128/mBio.03128-20 (PMC7885118; doi:10.1128/mBio.03128-20)

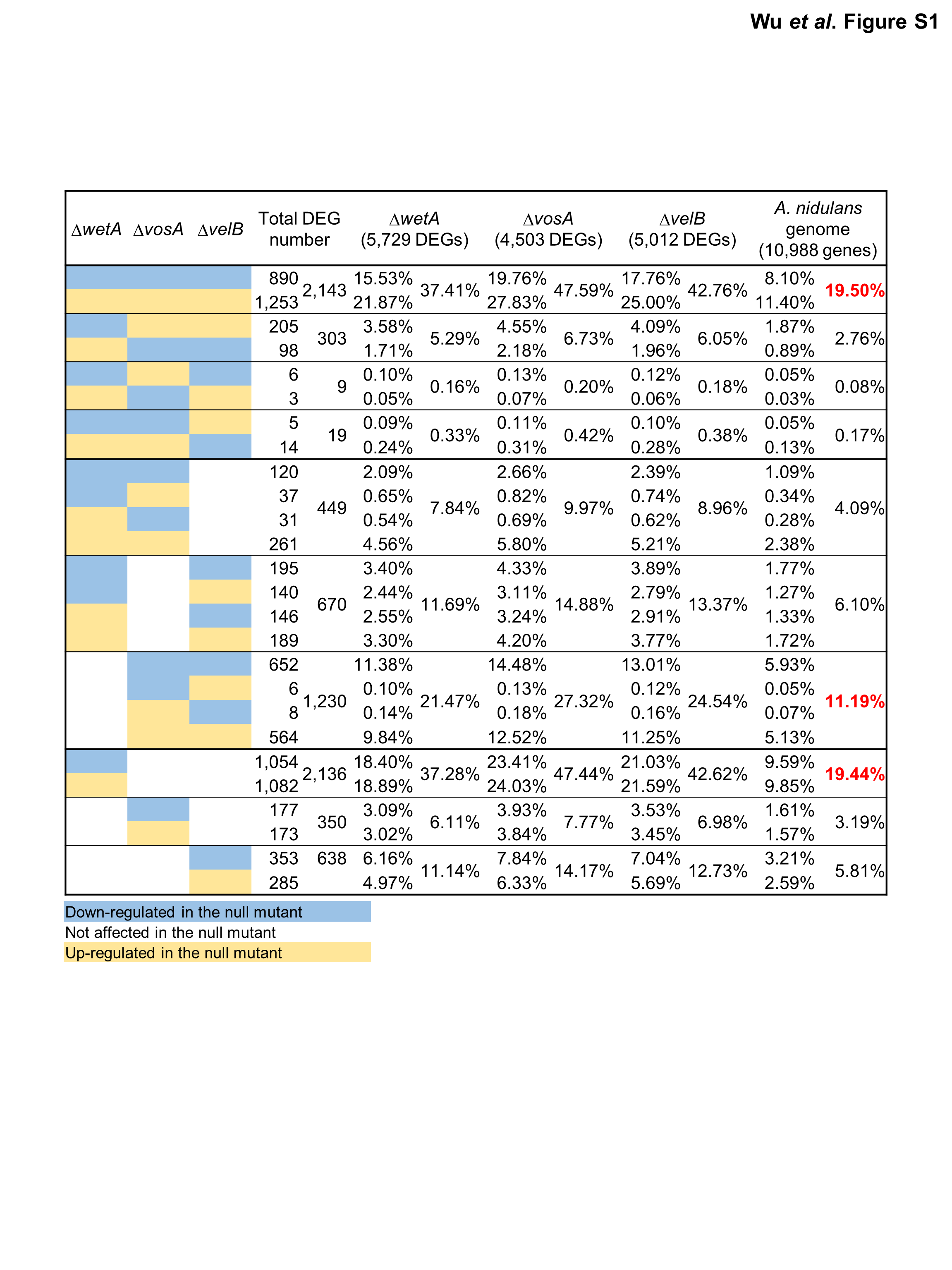

Supplement: FIG S1 [file mBio.03128-20-sf001.tif]

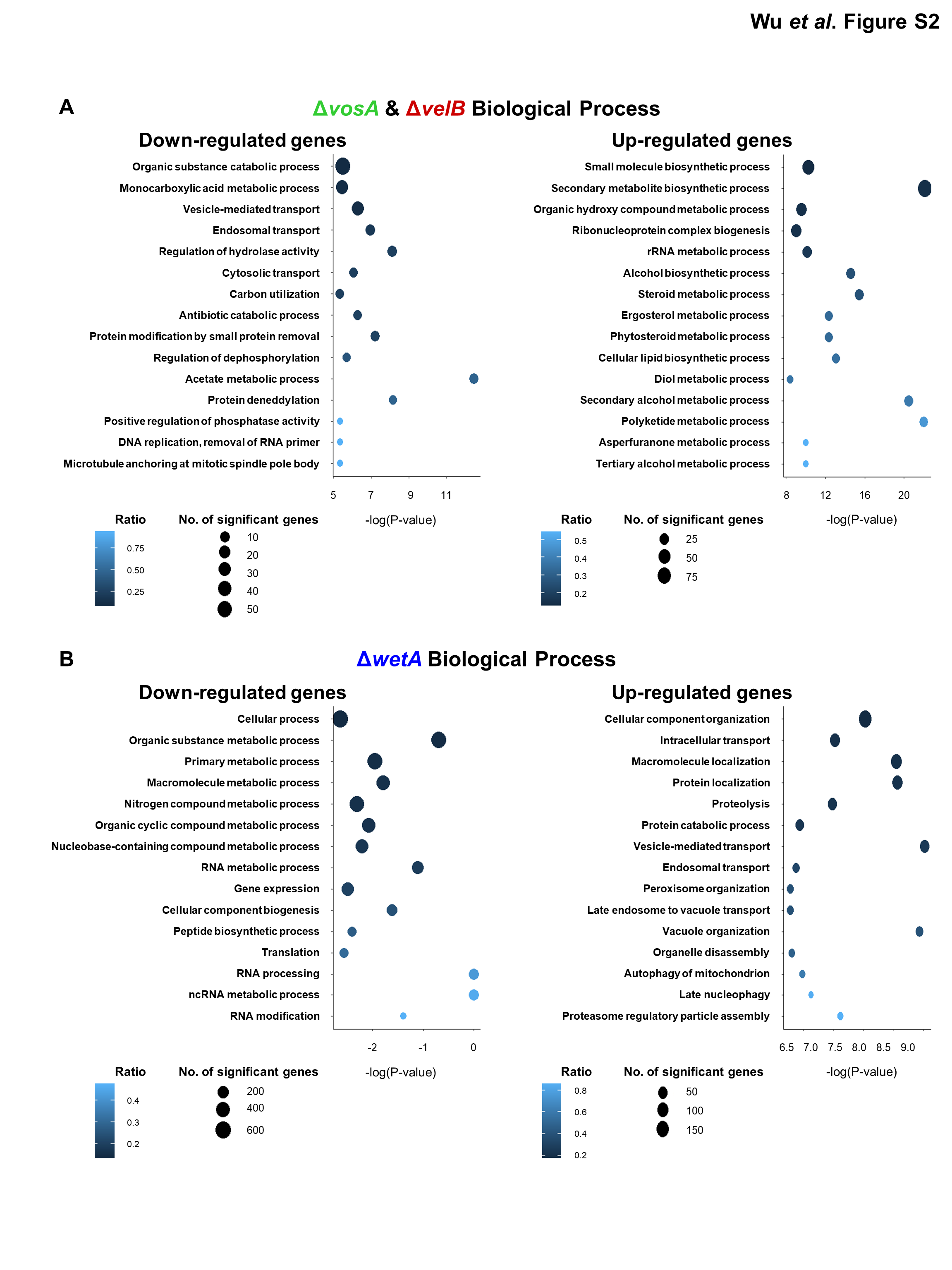

Supplement: FIG S2 [file mBio.03128-20-sf002.tif]

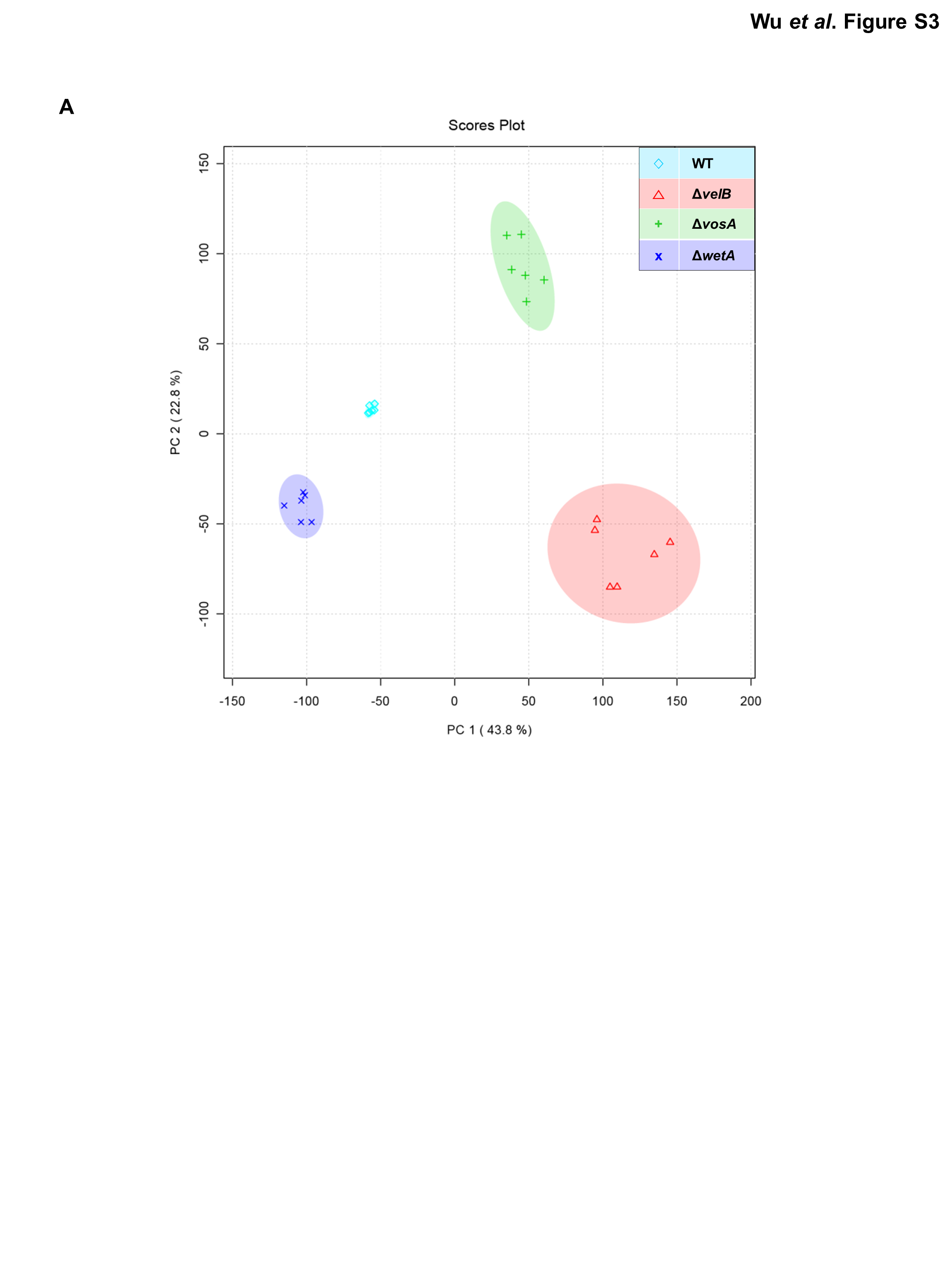

Supplement: FIG S3 [file mBio.03128-20-sf003.tif]
